# Calibrated and explainable multiparametric MRI radiomics for differentiating tumor positive disease from treatment-related changes in glioblastoma

**DOI:** 10.3389/fonc.2026.1890956

**Published:** 2026-08-18

**Authors:** Rafail C. Christodoulou, Georgios Vamvouras, Mateus A. Esmeraldo, Platon S. Papageorgiou, Evros Vassiliou, Elena E. Solomou, Sokratis G. Papageorgiou, Michalis F. Georgiou

**Affiliations:** 1 Division of Neuroimaging and Neurointervention, Department of Radiology, Stanford University, Stanford, CA, United States; 2 Department of Electrical and Computer Engineering, National Technical University of Athens (NTUA), Athens, Greece; 3 Department of Radiology, Stanford University, Stanford, CA, United States; 4 Department of Medicine, National and Kapodistrian University of Athens, Athens, Greece; 5 Department of Biological Sciences, Kean University, Union, NJ, United States; 6 Internal Medicine-Hematology, University of Patras Medical School, Rion, Greece; 7 1st Department of Neurology, Medical School, National and Kapodistrian University of Athens, Eginition Hospital, Athens, Greece; 8 Department of Radiology, University of Miami, Miami, FL, United States

**Keywords:** explainability (XAI), glioblastoma, machine learning, multiparametric MRI (mpMRI), radiomics, treatment-related changes, tumor positive disease

## Abstract

**Introduction:**

Differentiating tumor from treatment-related changes (TRC) remains a key challenge in post-treatment glioblastoma (GBM) monitoring, as both can exhibit overlapping enhancement and FLAIR abnormalities on standard MRI. Our goal was to build and internally validate an explainable, calibrated, multiparametric MRI radiomics model to distinguish between tumor and treatment effects.

**Methods:**

We retrospectively included patients from the UCSD-PTGBM cohort who had post-treatment GBM MRI examinations with available reference labels for tumor-positive disease (TP) or TRC. Examinations were eligible when contrast-enhanced T1-weighted imaging, FLAIR, ADC, DSC perfusion imaging, and corresponding tumor segmentation masks were available. After applying the eligibility and exclusion criteria, the final study cohort comprised 168 patients with 224 MRI examinations, 173 TP and 51 TRC cases. Radiomic features were extracted from all four MRI sequences. Feature selection and hyperparameter tuning were performed exclusively on the training data before evaluating the model on the test set. Performance metrics, calibration, decision curve analysis, and SHAP-based interpretability were assessed.

**Results:**

The extra trees classifier trained on the top 50 radiomic features demonstrated strong performance on the held-out test set, with an AUC of 0.887, PR-AUC of 0.952, balanced accuracy of 83.7%, sensitivity of 75.0%, and specificity of 92.3%. Platt calibration improved the Brier score from 0.229 to 0.132 and the expected calibration error from 0.275 to 0.084. Decision curve analysis indicated a net benefit across relevant clinical thresholds. SHAP analysis revealed balanced contributions from ADC, DSC, T1CE, and FLAIR modalities.

**Discussion:**

A calibrated and explainable multiparametric MRI radiomics model showed robust internal performance in differentiating tumor from treatment effects in post-treatment GBM. As these findings are hypothesis-generating from an internally validated study, external validation is needed to confirm clinical usefulness and broader applicability.

## Graphical Abstract

**Figure:**
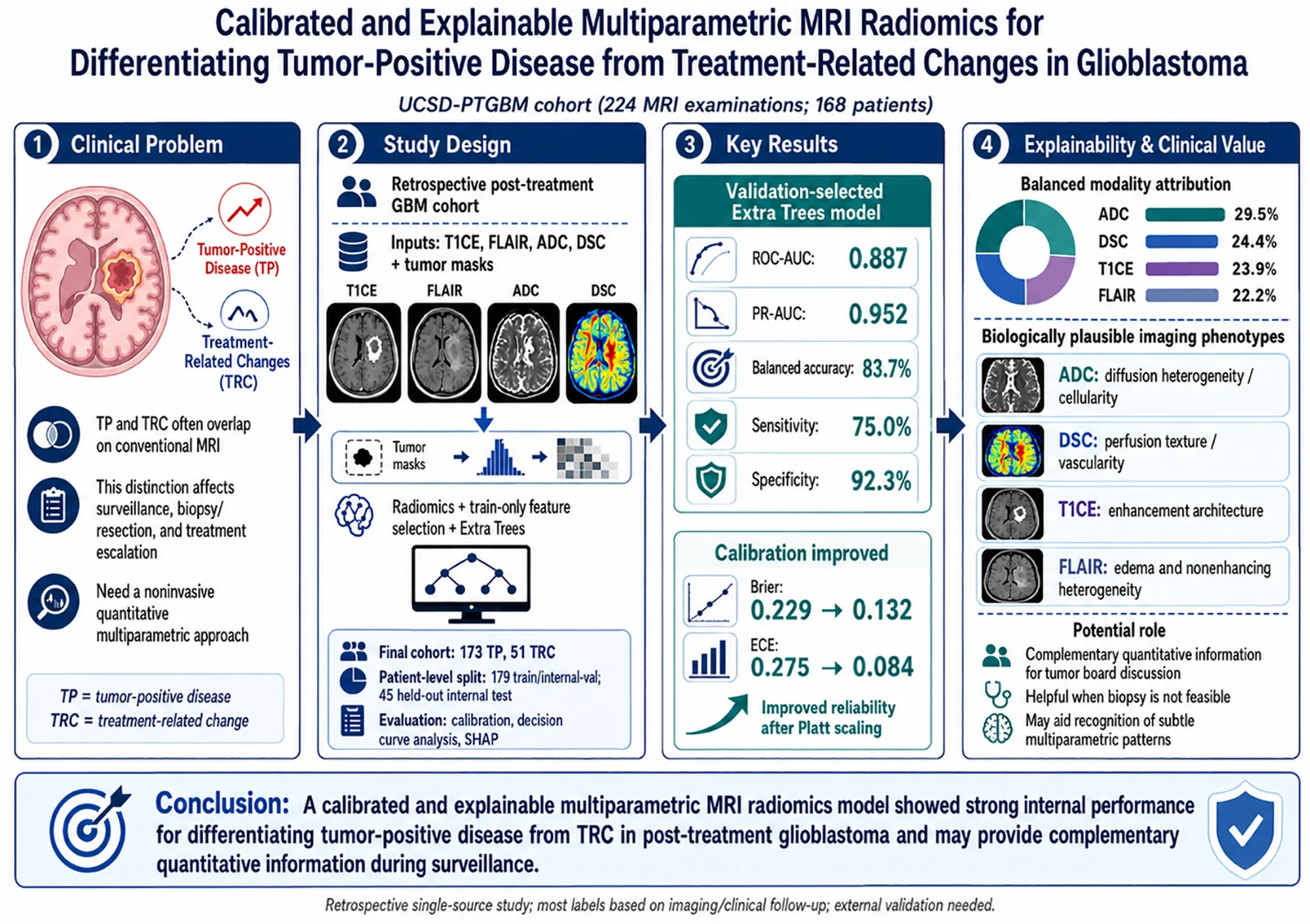

## Introduction

GBM is the most common malignant primary brain tumor in adults (1). Despite advances in neuroimaging, molecular biology, and genomics, overall survival (OS) remains low. Specifically, the median OS is approximately 15 months, while the 5-year OS is less than 5% (2, 3). Standard management involves performing the maximum safe resection, then administering radiotherapy together with temozolomide during and after treatment. This approach has established combined chemoradiation as the primary treatment strategy for newly diagnosed GBM (4).

Conventional MRI scans performed between 3–6 months post-treatment may show contrast enhancement, indicating disruption of the blood-brain barrier. The enhancement could be due to a recurrent tumor or TRC. Both have an almost identical appearance on conventional MRI sequences, making them difficult to differentiate (5). The distinction between the two conditions is vital, as it ultimately affects patient management, since TRC may indicate treatment response, tumor control, and therefore a better prognosis. On the other hand, a recurrent tumor indicates treatment failure and the need for treatment plan readjustment (6). Recent automated segmentation methods, such as nnU-Net, enhance the standardized assessment of postoperative tumor compartments by accurately segmenting glioblastoma components, including enhancing tumor, edema, and resection cavity (7). Standard anatomical MR imaging remains the primary method for response assessment, but it has inherent limitations in this context. Contrast enhancement primarily reflects blood-brain barrier disruption rather than tumor cell density, and FLAIR abnormalities can indicate infiltrative tumor, edema, treatment effects, or a combination of these factors (8). Advanced imaging methods, especially perfusion and diffusion-weighted MRI, can offer additional biological insights beyond standard MRI. Perfusion indicates microvascularity, while diffusion reflects tissue cellularity. DSC perfusion has demonstrated promising diagnostic accuracy in distinguishing glioma recurrence from radiation-induced changes, although differences in acquisition settings and thresholds continue to hinder standardization (9–11). The Response Assessment in Neuro-Oncology criteria were developed to standardize response assessment in gliomas, given these challenges. However, even with updated frameworks, interpreting early post-treatment enhancement remains difficult. There is also a need to combine imaging changes with factors such as timing, corticosteroid use, clinical condition, and follow-up observations (12). The definitive reference standard for differentiating TP from TRC is histopathologic confirmation obtained through surgical biopsy or resection. However, surgery is invasive, carries risks of morbidity, and may not be feasible or appropriate for all patients (13). Therefore, a reliable noninvasive imaging-based method could help guide management while reducing unnecessary surgical intervention.

Radiomics offers a quantitative approach to extracting high-dimensional imaging features that may capture tumor phenotypes beyond visual interpretation (14, 15). In neuro-oncology, radiomic and radiogenomic techniques are employed to analyze tumor biology, molecular characteristics, prognosis, and treatment outcomes from MR imaging (16, 17). For example, a recent meta-analysis highlighted the significance of MRI-based radiomics in predicting IDH mutation, with a pooled sensitivity of 86.7%. It also emphasizes its importance in patient management and clinical diagnosis (18). Notably, in glioma treatment outcomes, radiomics has been investigated to differentiate recurrent tumors from TRC. Kocak et al. (2025) demonstrated, in a radiomics-based meta-analysis, strong diagnostic capabilities for differentiating radiation-induced brain injury from glioma recurrence, with an estimated pooled AUC of 0.832. However, the authors highlighted significant heterogeneity, potential publication bias favoring positive results, and less-than-ideal methodological quality, which restricts its current clinical application (19). Several translational aspects remain inconsistently reported, including probability calibration and clinical utility assessment (19).

Further research is needed to assess whether radiomics can reliably distinguish active tumors from TRC and if model outputs are trustworthy, interpretable, and useful in clinical settings. We hypothesized that multiparametric MRI radiomics could distinguish TP from TRC in post-treatment GBM, providing clear, clinically relevant risk assessments. To test this, we developed and validated six machine learning-based radiomic features from T1CE, FLAIR, ADC, and DSC, and implemented probability calibration, decision curve analysis, and SHAP-based explanations at the modality and feature levels.

## Materials and methods

### MRI data acquisition and study cohort

MRI data were obtained from the post-treatment glioblastoma cohort in the UCSD-PTGBM database (20). This retrospective dataset included MRI examinations from patients with treated GBM and available reference labels indicating either recurrent/residual tumor as TP or TRC. Tumor was defined as the positive class, because failure to identify tumor in practice represents a higher-risk clinical error. Examinations were eligible when contrast-enhanced T1-weighted imaging, FLAIR, ADC, and DSC perfusion imaging were available, together with corresponding tumor segmentation masks.

Before modeling, 19 TP examinations from 16 patients were excluded to reduce potential confounding: 9 examinations were excluded because bevacizumab had been administered within 28 days of MRI (21), and 10 examinations were excluded because they involved IDH-mutant tumors with available ADC/ASL-family imaging, given reported diffusion and perfusion differences between IDH-mutant and IDH-wildtype gliomas that could confound diffusion and perfusion-based classification (22). Some of these patients contributed other eligible examinations that remained in the analysis. After these exclusions, the final multimodal cohort included 224 MRI examinations from 168 patients, comprising 173 TP and 51 TRC. Data were split at the patient level to prevent leakage from repeated examinations. The training/internal-validation set included 179 examinations from 134 patients, and the held-out internal test set included 45 examinations from 34 patients. No patient contributed to both sets of examinations. The final cohort characteristics are as described in **Table 1**. A full flow cohort is available in the supplement(**Supplementary Figure 1**).

**Table 1:** Baseline patient characteristics stratified by reference-standard diagnosis (tumor-positive disease [TP] vs. treatment-related change [TRC]). Values are reported at the patient level unless otherwise indicated. P values compare TP vs. TRC groups using Welch's t-test (age) or Fisher's exact test (categorical variables).

Characteristic	Overall	TP	TRC	Test	P value
Patients, n	168	139	29		
Imaging examinations, n	224	173	51		
Age at first included scan, years	56.3 ± 13.1	57.7 ± 12.3	49.2 ± 14.7	Welch t test	0.006
Sex				Fisher exact	1.000
Male	123 (73.2%)	102 (73.4%)	21 (72.4%)		
Female	45 (26.8%)	37 (26.6%)	8 (27.6%)		
MGMT promoter status	100	80	20	Fisher exact	0.002
Methylated	49 (49.0%)	33 (41.3%)	16 (80.0%)		
Unmethylated	51 (51.0%)	47 (58.8%)	4 (20.0%)		

Values are reported at the patient level unless otherwise indicated. Percentages for MGMT promoter status are calculated among patients with available MGMT data.

### Reference standard

Ground-truth labels were derived from the UCSD-PTGBM database and assigned at the MRI examination level. In the original dataset, post-treatment MRI time points were categorized as residual/recurrent tumor or post-treatment change based on clinical, radiological, and pathological data, including RANO-based assessments and longitudinal follow-up. Investigators reviewed the pathology database to add progression- and treatment-related cases confirmed by pathology to the cohort. In this study, exams labeled as residual or recurrent tumor were grouped as the positive class(TP), while post- treatment change was categorized as negative, including pseudoprogression, radiation necrosis, and nonspecific treatment effects. This classification follows the UCSD-PTGBM binary standard to differentiate tumor-positive disease from isolated treatment effects. Although residual tumor, recurrence, and progression are biologically distinct, all are treated as tumor-positive in the labels and are separated from treatment-related changes, which are clinically distinct. This grouping reflects the core clinical question: whether an abnormality indicates tumor-positive disease requiring active management or an isolated treatment effect suitable for observation or continuation of therapy. Follow-up duration was from the initial MRI date to the last available follow-up. For exams with follow-up data, the median follow-up was 266.5 days for TP and 551 days for TRC. Labels were assigned at the exam level, although some patients contributed multiple exams; patient- level splitting was used during model training to avoid data leakage. Six treatment- related exams from four patients had biopsy or pathology confirmation. While the original dataset included pathology-confirmed progression timepoints, the archived metadata lacked a reliable per-examination pathology confirmation flag in the final cohort after exclusions. 

### MRI preprocessing and segmentation

Tumor masks were derived from the UCSD-PTGBM/BraTS-style annotations provided in the dataset. According to the dataset documentation, multicompartment segmentations were initially created using automated deep-learning software (OnQ Neuro; Cortechs.ai, San Diego, CA, USA) and then manually refined by trained radiologists. These annotations included categories such as enhancing tumor, nonenhancing/necrotic tumor, surrounding FLAIR abnormality, and resection cavity. For radiomic analysis, all nonzero tumor compartment labels were binarized into a single whole-lesion region of interest (ROI). The same ROI was used across T1CE, FLAIR, ADC, and DSC images; no sequence-specific or compartment-specific ROIs were created. This lesion-level approach was chosen to capture multiparametric heterogeneity within the entire post-treatment abnormality. Radiomic features were extracted from the final binary ROI with PyRadiomics. During preprocessing, image-mask geometry was verified, and overlays were visually inspected for proper alignment and coverage before feature extraction. All images—T1CE, FLAIR, ADC, and DSC—were resampled to the binary lesion mask grid when necessary, with a target isotropic spacing of 1 × 1 × 1 mm. Resampling used SimpleITK B-spline interpolation for images and nearest-neighbor interpolation for masks. DSC radiomic features were obtained from leakage-corrected cerebral blood volume maps provided in the dataset, rather than from the raw DSC signal time series. Intensity normalization was performed using z-scores within the whole-lesion ROI, based on the mean and standard deviation of voxels within the tumor mask. PyRadiomics normalization was also applied with a scale of 100. Gray-level discretization employed a fixed bin width of 25 across all sequences and maps to ensure consistency. Since the study used dataset-provided final masks, independent reader-specific masks were unavailable, preventing inter-reader Dice coefficient calculations and assessments of segmentation robustness.

### Radiomic feature extraction

Radiomic features were obtained using PyRadiomics. For each scan, features were extracted separately from T1CE, FLAIR, ADC, and DSC images within the region of interest. Features were derived from original, Laplacian-of-Gaussian-filtered, and wavelet-filtered images, with Laplacian-of-Gaussian filtering applied at sigma values of 0.5, 1.0, 2.0, and 3.0 mm, using a fixed bin width of 25. The radiomic feature set included various feature families such as shape, first-order intensity, gray-level co-occurrence, run-length, size-zone, dependence, and neighboring gray-tone difference matrices. In total, 4892 features were extracted across four MRI sequences. The extraction process and statistical analysis were conducted in Python 3.12.1, utilizing PyRadiomics 3.1.1, SimpleITK 2.5.4, NumPy 2.4.4, pandas 3.0.2, and scikit-learn 1.8.0.

### Radiomic feature ranking and redundancy reduction

The radiomic features were split into a training/internal-validation set and an independent test set. To avoid patient-level leakage, the division used unique patient identifiers rather than individual exams. All exams from the same patient were assigned exclusively to either the training or test set. All preprocessing steps, feature filtering, ranking, hyperparameter tuning, thresholding, and model selection were performed only within the training set. Missing values were imputed using medians from training data, and features with near-zero variance were removed. Redundant features were filtered out based on Spearman correlation coefficients greater than 0.80, with thresholds tested during model development. Feature ranking was conducted using a train-only approach, incorporating coefficients from regularized logistic regression and permutation importance, and evaluated via grouped cross-validation to prevent leakage from repeated exams. The final feature ranking informed the selection of top-K feature subsets for modeling. 

To assess the impact of redundancy removal, absolute Spearman correlation matrices were generated before and after filtering. These matrices were summarized at the radiomic family level by calculating mean absolute correlations within and between families. Redundancy was further evaluated by the 95th percentile of absolute correlations and the percentage of feature pairs exceeding |ρ| > 0.8.

### Repeated examinations and patient-level analysis

The final cohort included 168 patients and 224 MRI examinations. Forty-two patients (25.0%) contributed more than one examination, with a median of 1 examination per patient (IQR, 1–1.25; range, 1–4). Ten patients had examinations with discordant labels over time, reflecting disease evolution; these patients were retained, and each examination was labeled independently according to its corresponding reference standard. All data partitioning and cross-validation were performed at the patient level. The cohort was divided into training/validation (134 patients, 179 examinations) and test (34 patients, 45 examinations) sets with no patient overlap. Cross-validation used a 5-fold StratifiedGroupKFold scheme, grouped by patient ID to ensure that all examinations from a single patient remained in the same fold. Model performance, calibration, decision curve analysis, and threshold optimization were evaluated at the examination level, as each examination constitutes an independent clinical input to the model. To account for within-patient correlation arising from patients who contributed multiple examinations, bootstrap confidence intervals were estimated with resampling clustered at the patient level.

### Model Development and hyperparameter optimization

Model development involved splitting patients into training/validation and test cohorts. All feature ranking, filtering, hyperparameter tuning, threshold selection, and calibration fitting used only the training/validation cohort. The test set was not used for these steps. Feature ranking employed a 10-fold StratifiedGroupKFold based on patient ID, combining L1-regularized logistic regression coefficients with permutation importance. Redundancy filtering within training/validation used Spearman correlation, reducing 4892 features to 463. Candidate top-K features included 25, 50, 100, 200, 300, and 463. Main model tests evaluated elastic-net logistic regression, RBF support vector machines, and Extra Trees classifiers; other classifiers were benchmarked. To address class imbalance, evaluated classifiers used class-weighted learning, including class_weight="balanced" for elastic-net logistic regression, RBF-SVM/SVC, Extra Trees, and histogram gradient boosting, and class_weight="balanced_subsample" for random forest. Hyperparameters were optimized with Optuna (seed 42, 25 trials per model/K, 5-fold StratifiedGroupKFold). The objective was ROC-AUC on out-of-fold predictions. Cross-validation kept all exams for the same patient in a single fold. Thresholds for training/out-of-fold predictions were selected using the Youden index, with TP as the positive class. Calibration was also fitted using these predictions. The Extra Trees model with K=50 was selected after benchmarking.

### Model evaluation

After selecting the model within the training/internal-validation set, the chosen multimodal model was tested on an independent, held-out test set. Its discrimination ability was primarily measured using ROC-AUC, with additional metrics including precision-recall AUC, Brier score, accuracy, balanced accuracy, sensitivity for TP, specificity for treatment-related change, positive and negative predictive values, F1 score, and Matthews correlation coefficient. Confidence intervals were estimated through patient-cluster bootstrap resampling to account for multiple examinations per patient. Threshold-dependent metrics were determined using the Youden threshold from the training set. Calibration was evaluated with the Brier score, log loss, and expected calibration error. Platt scaling and isotonic calibration were tested on out-of-fold training predictions before being applied to the test set. Finally, clinical utility was assessed via decision curve analysis, comparing net benefit across different threshold probabilities relative to treat-all and treat-none strategies.

### Explainability

Model explainability was assessed using SHAP values. SHAP contributions were summarized at the individual feature, feature family, and imaging modality levels (23). For modality-level interpretation, mean absolute SHAP values were aggregated across features originating from the same MRI sequence. This enabled estimation of the relative contribution of ADC, DSC, T1CE, and FLAIR features to the model’s predictions. Feature-family-level analysis was performed by aggregating SHAP values across radiomic families, including first-order, GLCM, GLRLM, GLSZM, GLDM, and NGTDM features. A cumulative SHAP importance curve was generated to evaluate whether model attribution was distributed broadly across the feature set or concentrated among a smaller subset of dominant predictors.

## Results

### Radiomic feature reduction and redundancy structure

**Figure 1:**
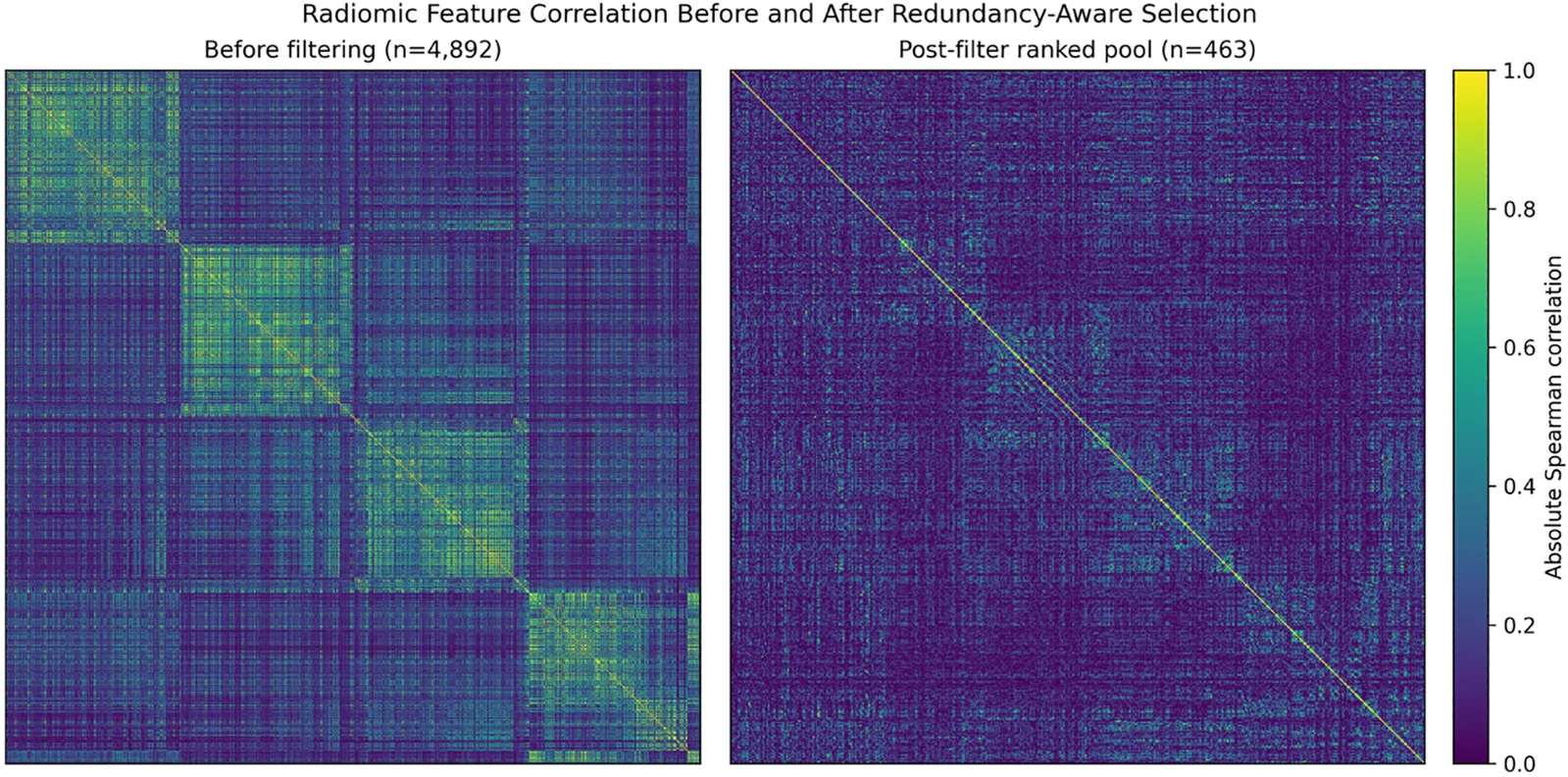
Radiomic feature correlation before and after redundancy-aware selection. Absolute Spearman correlation matrices before filtering are shown for the full radiomic feature set (n = 4892) and after redundancy-aware selection for the post-filter ranked pool (n = 463). Dense high-correlation blocks before filtering are markedly reduced after filtering, indicating effective attenuation of redundant radiomic descriptors.

The independent held-out test set included 45 post-treatment glioblastoma MRI examinations from 34 patients, comprising 32 TP and 13 treatment-related-change examinations. Across T1CE, FLAIR, ADC, and DSC imaging, 4892 radiomic features were extracted. After train-only feature ranking and redundancy reduction within the training/internal-validation set, the candidate feature pool was reduced to 463 ranked features. The validation-selected multimodal model was an Extra Trees classifier using the top 50 ranked radiomic features. Redundancy-aware feature selection substantially reduced inter-feature correlation. Before filtering, the absolute Spearman correlation matrix demonstrated dense blocks of highly correlated radiomic descriptors, reflecting substantial redundancy among features generated from related filters, modalities, and texture families. After filtering, the post-filter ranked pool showed marked attenuation of these high-correlation blocks, indicating a less collinear feature space while preserving broad radiomic feature diversity (**Figure 1**).

**Figure 2:**
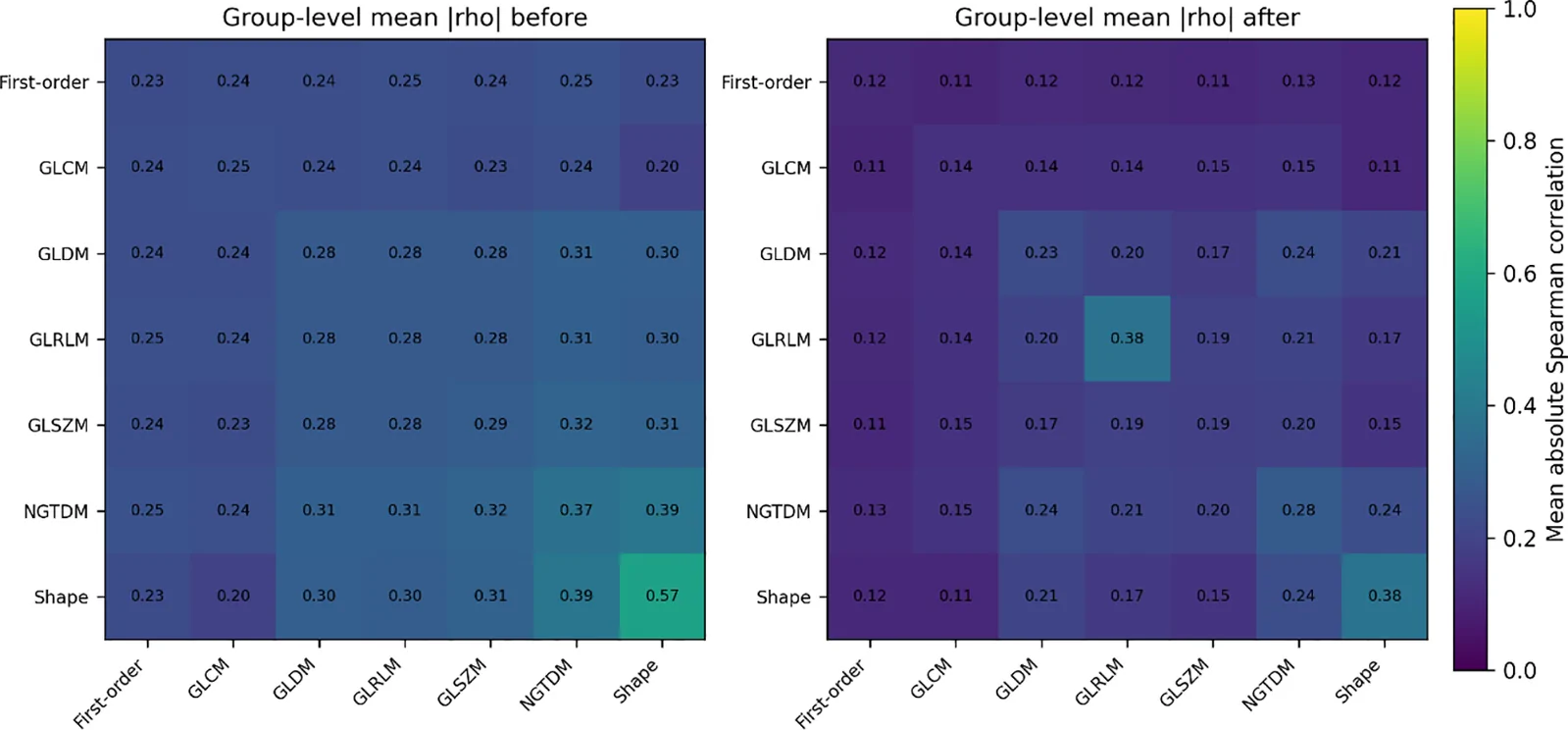
Group-level mean absolute Spearman correlation before and after filtering. Heatmaps show mean absolute Spearman correlation values within and between radiomic feature families before and after redundancy-aware filtering. After filtering, most family-level correlation blocks show lower mean |ρ|, indicating reduced redundancy among retained descriptors.

At the radiomic family level, mean absolute correlations were reduced across nearly all within-family and between-family blocks after filtering. Before filtering, higher mean correlations were observed within texture-based families and between related texture groups, including GLDM, GLRLM, GLSZM, NGTDM, and shape-associated descriptors. After filtering, intra-family and cross-family correlations decreased, supporting the effectiveness of redundancy reduction at both individual-feature and descriptor-family levels (**Figure 2**).

**Figure 3:**
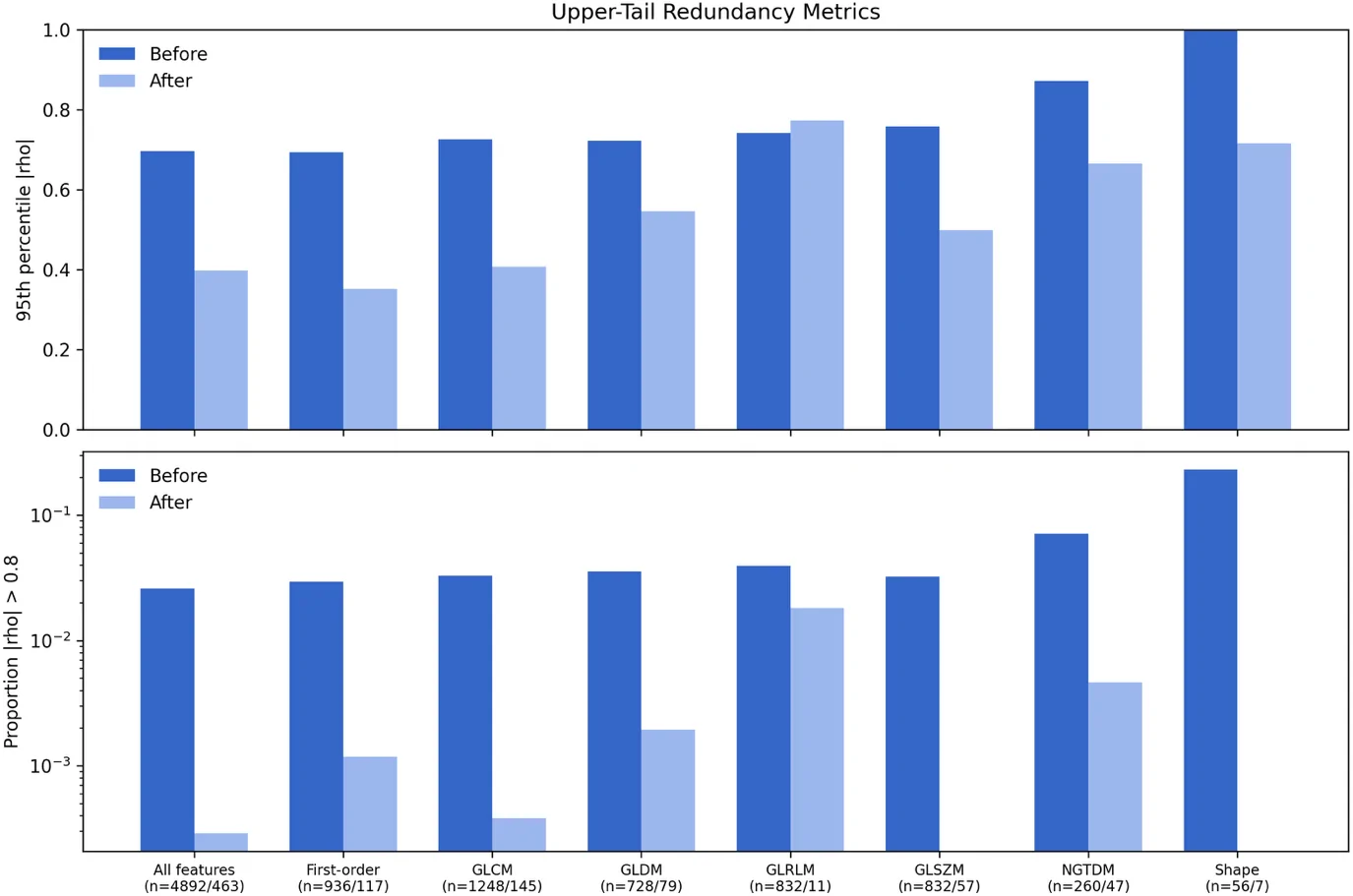
Upper-tail redundancy metrics before and after filtering. The upper panel shows the 95th percentile of absolute Spearman correlation before and after filtering. The lower panel shows the proportion of feature pairs with |ρ| > 0.8 on a logarithmic scale. Redundancy-aware filtering reduced both extreme pairwise correlations and the fraction of strongly correlated feature pairs.

Upper-tail redundancy metrics further confirmed the effect of filtering. The 95th percentile of absolute Spearman correlation decreased across the overall feature set and most feature families. The proportion of strongly correlated feature pairs with |ρ| > 0.8 also decreased markedly after filtering, indicating preferential removal of near-duplicate descriptors (**Figure 3**).

### Modality composition across ranked features

The composition of the ranked feature pool varied across top-K feature subsets. Among the highest-ranked features, ADC-derived descriptors initially contributed the most, making up about 40% of the selected features at very low K values. As K grew, T1CE-derived features became more dominant, reaching roughly 50% of the selected features around K ≈ 160. FLAIR-derived features increased later, peaking at approximately 40% near K ≈ 250. DSC-derived features played a smaller role at lower and middle K values but became more prominent in larger feature sets.

**Figure 4:**
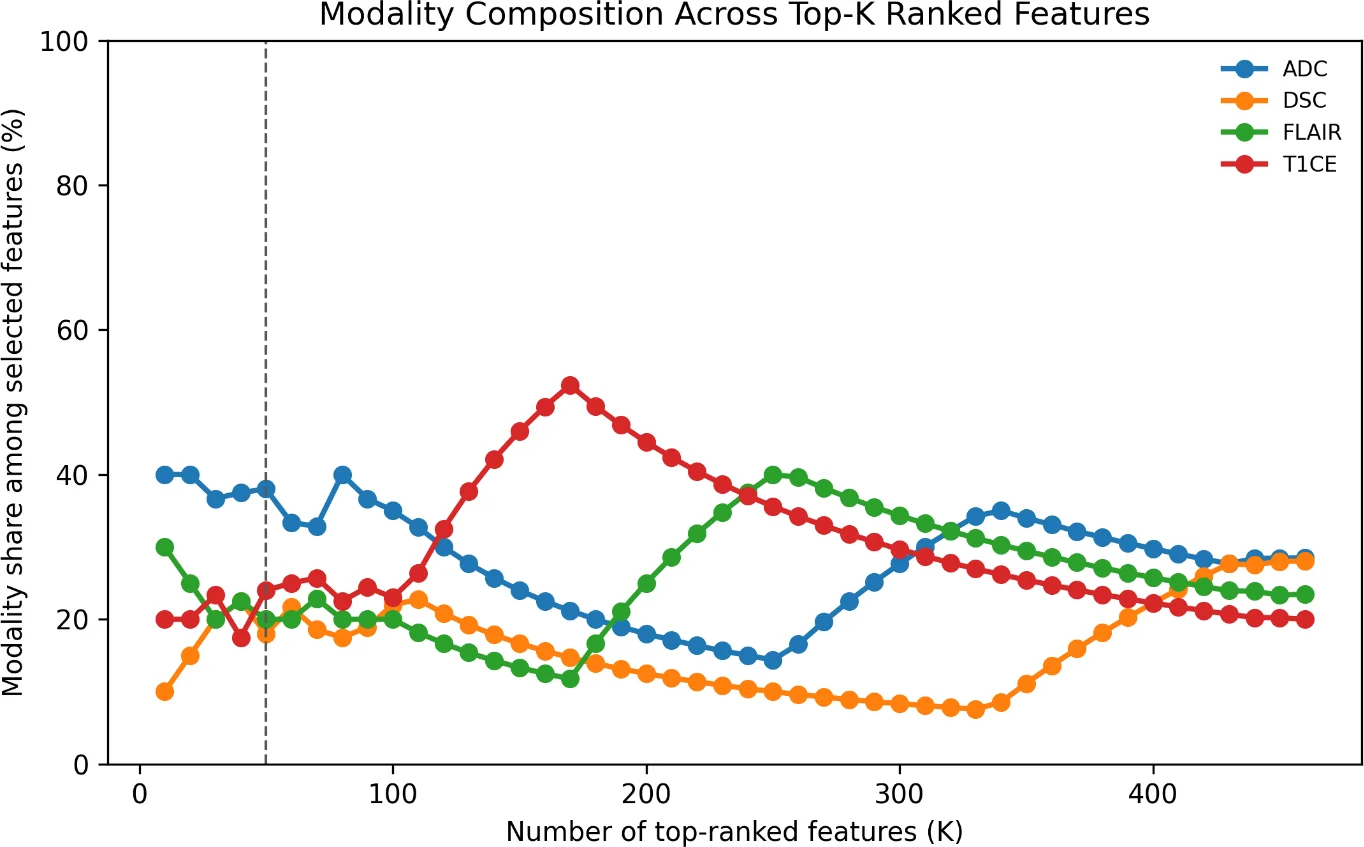
Modality composition across top-K ranked features. The relative percentages of ADC, DSC, FLAIR, and T1CE features are shown as increasingly larger top-K subsets are sampled from the ranked feature list. The vertical dashed line indicates the selected **K** **=** **50** configuration used for model development. At K = 50, all four modalities contribute to the selected feature subset, supporting the multiparametric composition of the final feature pool. Trends beyond the selected K are shown descriptively to illustrate how modality representation changes across the ranking spectrum.

At K = 50, marked by the vertical reference line, the top features included all four modalities, indicating that the validation-selected model used multiparametric information rather than relying on a single MRI sequence (**Figure 4**).

### Classification performance

**Figure 5:**
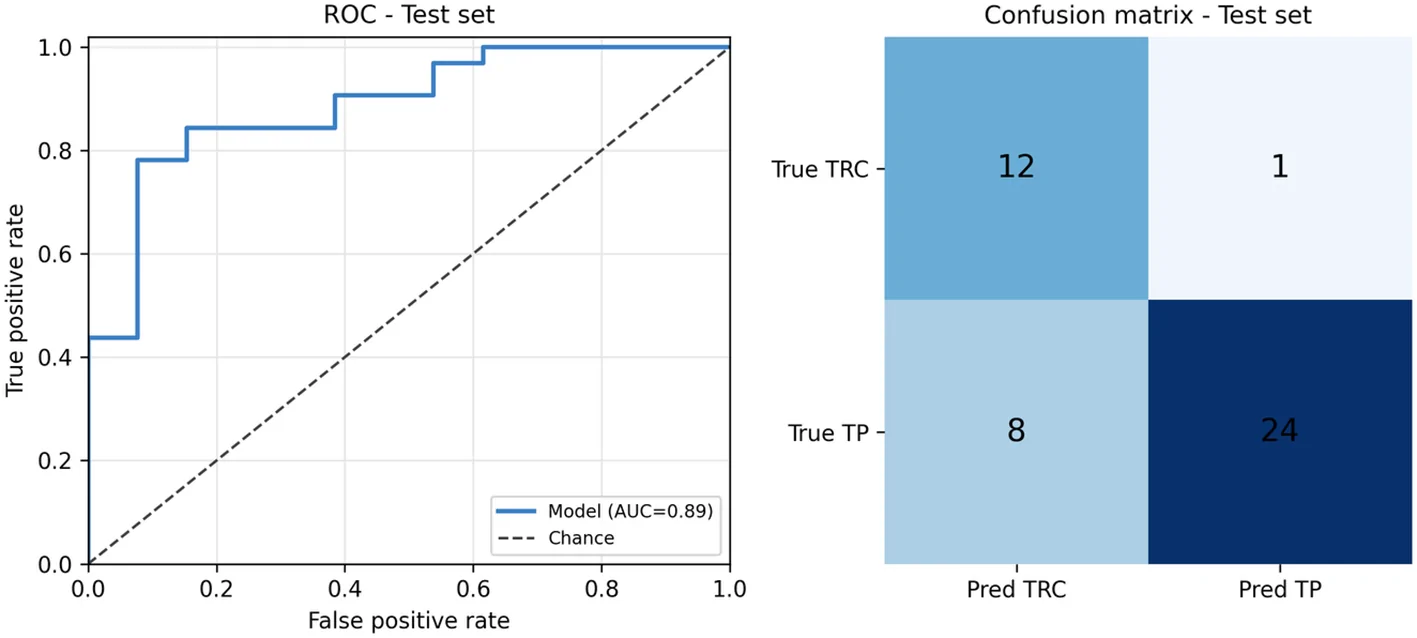
Held-out test ROC curve and confusion matrix for the Extra Trees model. The ROC curve demonstrates strong held-out discrimination, with an AUC of approximately 0.89. The confusion matrix shows 24 true positives, 12 true negatives, 1 false positive, and 8 false negatives using the training-derived Youden threshold.

The Extra Trees model,on the independent test set, the model achieved an ROC-AUC of 0.887 (95% patient-cluster bootstrap CI, 0.765–0.972) and a precision-recall AUC of 0.952 (95% CI, 0.881–0.992) at a TP of 0.711. The uncalibrated Brier score was 0.229. A threshold based on training/internal-validation out-of-fold predictions and the Youden index was used to balance sensitivity and specificity without relying on held-out test data. This yielded an accuracy of 80.0%, a balanced accuracy of 83.7%, a sensitivity of 75.0% for TP, and a specificity of 92.3% for treatment-related change. The F1 score for TP was 0.842, and the Matthews correlation coefficient stood at 0.614. The confusion matrix revealed 24 true positives, 12 true negatives, 1 false positive, and 8 false negatives (**Figure 5**).

Held-out test benchmarking of additional internally evaluated configurations is shown in **Table 2**. These results are presented descriptively to contextualize the selected model’s performance and were not used to select the primary model after test-set evaluation. The selected Extra Trees (K = 50) model achieved the highest held-out ROC-AUC among the listed configurations, while the RBF-SVM (K = 200) showed similar discrimination with a lower Brier score. Elastic-net logistic regression and random forest configurations also demonstrated competitive performance, supporting the robustness of the multimodal radiomic signal across classifier families. Lastly, to demonstrate the robustness of the selected model, we performed training-only bootstrap feature stability analysis and learning-curve analysis. Stability was stronger at the modality and feature-family levels than at the individual-feature level, and learning-curve analysis demonstrated increasing performance with larger training fractions, with relative plateauing between 80% and 100% of training patients (**Supplementary Figure 2–S3**).

**Table 2:** Test-set performance of internally evaluated classifier configurations.

Rank	Classifier	Features	Test AUC	95% CI	PR-AUC	Brier Score	Balanced Accuracy	TP Sensitivity	TRC Specificity	F1 Score	MCC
1	**Extra Trees**	50	0.887	0.765–0.972	0.952	0.229	0.837	0.750	0.923	0.842	0.614
2	RBF-SVM	200	0.885	0.754–0.976	0.946	0.135	0.806	0.844	0.769	0.871	0.589
3	Extra Trees	100	0.873	0.752–0.957	0.951	0.186	0.768	0.844	0.692	0.857	0.525
4	Elastic-net Logistic Regression	200	0.865	0.728–0.957	0.945	0.156	0.798	0.750	0.846	0.828	0.547
5	Elastic-net Logistic Regression	463	0.856	0.737–0.947	0.945	0.166	0.752	0.812	0.692	0.839	0.485
6	RBF-SVM	300	0.853	0.701–0.962	0.929	0.145	0.791	0.812	0.769	0.852	0.551
7	Random Forest	463	0.841	0.687–0.950	0.923	0.155	0.775	0.781	0.769	0.833	0.515
8	Random Forest	200	0.798	0.631–0.919	0.909	0.175	0.775	0.781	0.769	0.833	0.515
9	SVC	100	0.798	0.621–0.920	0.906	0.192	0.591	0.875	0.308	0.812	0.217
10	Histogram Gradient Boosting	220	0.772	0.578–0.908	0.885	0.166	0.674	0.656	0.692	0.737	0.318

Bold indicates the validation-selected primary model (Extra Trees, K = 50); other configurations are shown descriptively for comparison and were not used for model selection after test-set evaluation.

### Calibration

Probability calibration improved the reliability of predicted probabilities while preserving discrimination. Platt calibration maintained the held-out test ROC-AUC at 0.887 and improved the Brier score from 0.229 to 0.132. Expected calibration error also improved, decreasing from 0.275 before calibration to 0.084 after Platt calibration. Isotonic calibration yielded a slightly lower Brier score but reduced discrimination on the held-out test set. Therefore, Platt calibration remained the main calibration method (**Table 3**). It was chosen because it improved the Brier score and expected calibration error while maintaining discriminative ability, and offered a less flexible alternative to isotonic regression, suitable for the relatively small held-out test set. Threshold-dependent metrics for the selected model are summarized in **Table 4**.

**Table 3:** Probability calibration across the selected model.

Method	Test AUC	Brier score	95% CI	Log loss	ECE	95% CI
Uncalibrated	0.887	0.229	0.222–0.236	0.650	0.275	0.227–0.396
Platt calibrated	0.887	0.132	0.071–0.203	0.407	0.084	0.046–0.218
Isotonic sensitivity analysis	0.871	0.127	0.071–0.191	0.611	0.077	0.037–0.193

Confidence intervals were estimated using patient-cluster bootstrap resampling with 5000 resamples. Platt calibration was retained as the primary calibration method because it improved Brier score, log loss, and ECE while preserving discrimination. Isotonic calibration was treated as a sensitivity analysis due to the small held-out test set and the greater flexibility of isotonic regression.

**Table 4:** Threshold-dependent performance of the selected model on the held-out test set.

Metric	Estimate	95% CI
Sensitivity	0.750	0.590–0.886
Specificity	0.923	0.727–1.000
PPV	0.960	0.871–1.000
NPV	0.600	0.357–0.810
Accuracy	0.800	0.683–0.905
Balanced accuracy	0.837	0.726–0.926
F1 score	0.842	0.727–0.928
MCC	0.614	0.392–0.800

Metrics were calculated on the held-out test set of 45 examinations from 34 patients using the training-derived Youden threshold. Confidence intervals were estimated using patient-cluster bootstrap resampling with 5000 resamples.

### Decision curve analysis

**Figure 6:**
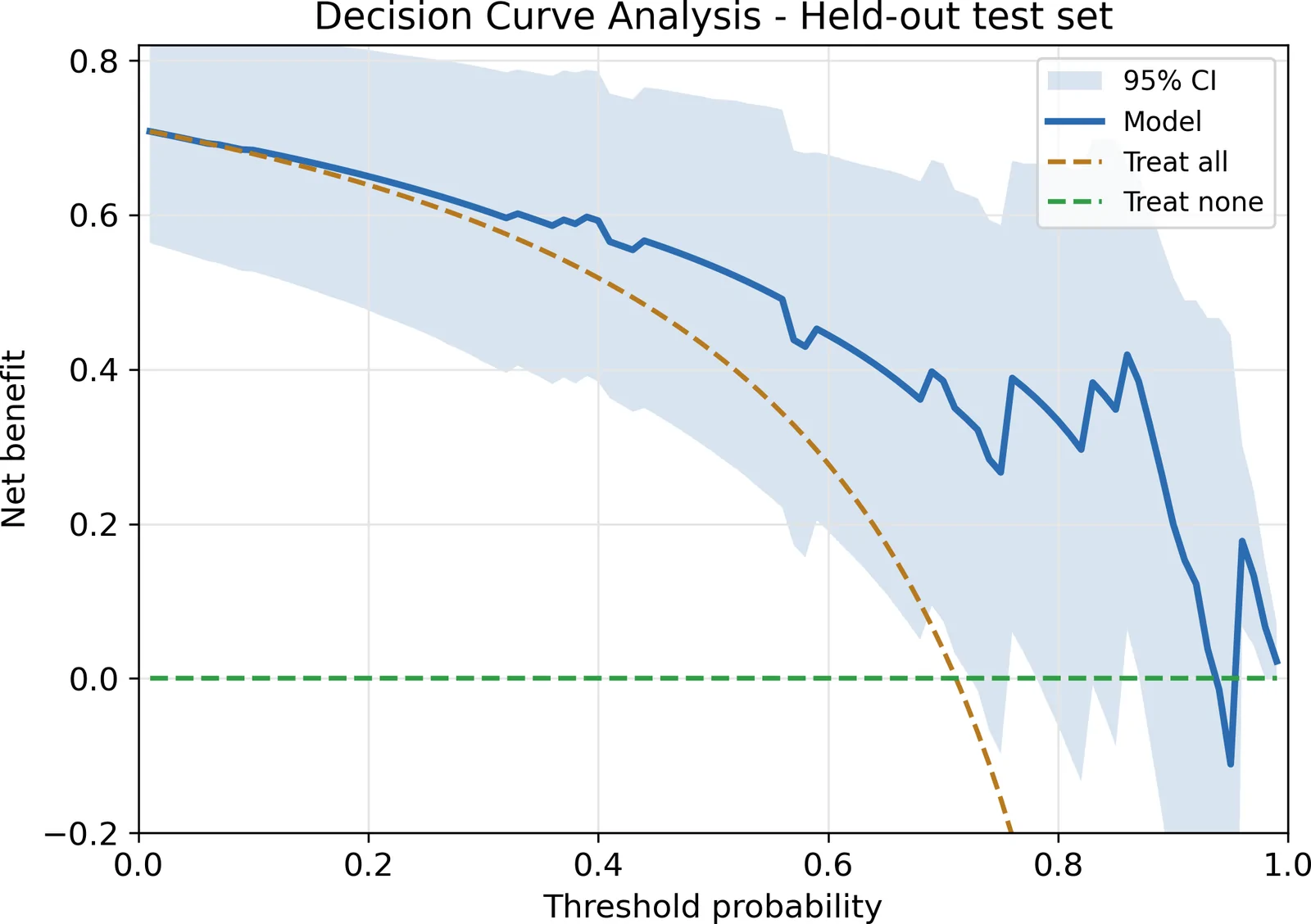
Decision curve analysis on the independent held-out test set. Net benefit is shown across threshold probabilities for the selected model compared with treat-all and treat-none strategies.

Exploratory decision curve analysis suggested that the validation-selected model may provide positive net benefit across low-to-intermediate threshold probabilities compared with treat-all and treat-none strategies. However, the confidence bands were wide, particularly at higher threshold probabilities, indicating substantial uncertainty in the net-benefit estimates. Therefore, these findings should be interpreted as preliminary evidence of potential clinical usefulness rather than definitive evidence of clinical utility. The instability at higher thresholds likely reflects the limited size of the held-out test set and the small number of treatment-related change examinations (**Figure 6**).

### Explainability

**Figure 7:**
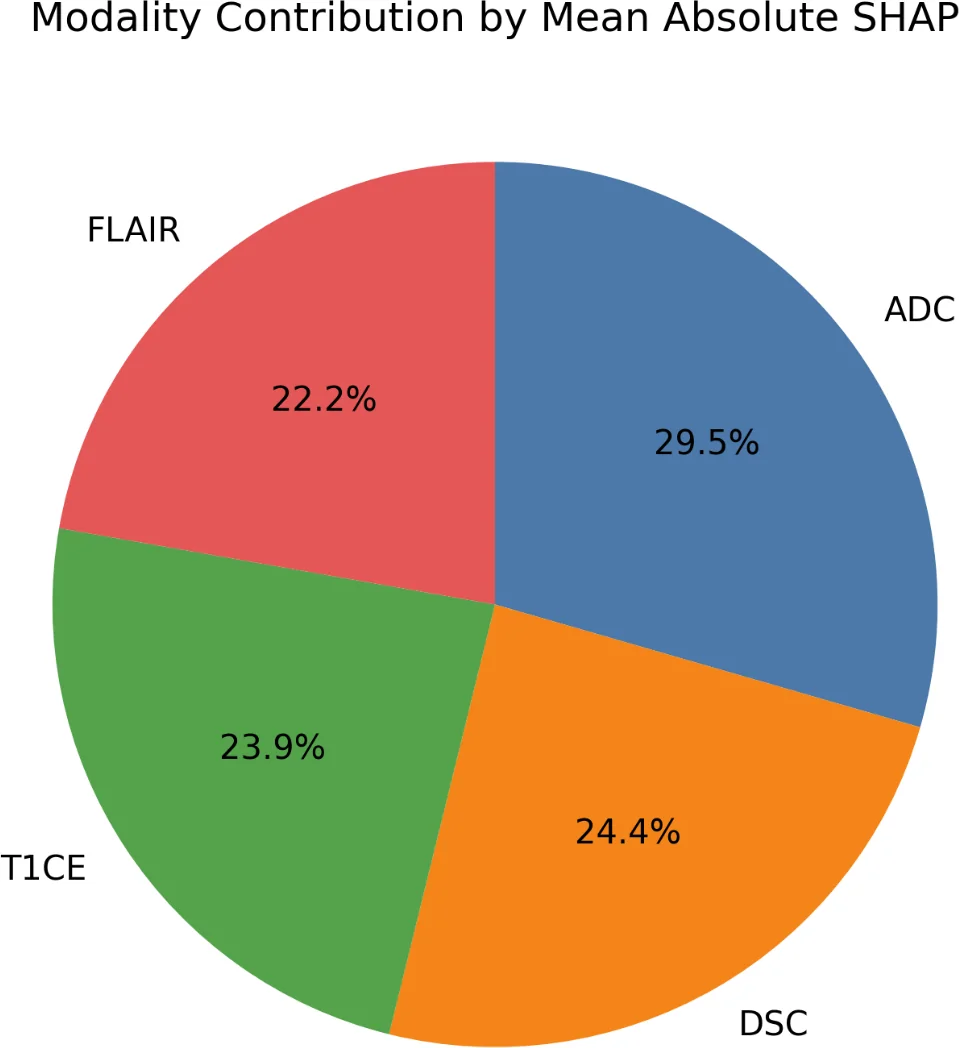
Modality contribution by mean absolute SHAP. Mean absolute SHAP values were aggregated by imaging modality. ADC contributed 29.5% of total attribution, followed by DSC at 24.4%, T1CE at 23.9%, and FLAIR at 22.2%, indicating balanced multimodal contribution across diffusion, perfusion, postcontrast, and FLAIR-derived radiomic information.

SHAP analysis revealed that the model’s predictions relied on all four MRI modalities rather than being dominated by one sequence. The contributions from each modality were ADC at 29.5%, DSC at 24.4%, T1CE at 23.9%, and FLAIR at 22.2%. These results suggest that radiomic features from diffusion, perfusion, postcontrast, and FLAIR imaging all played significant roles in the model’s decision-making process. ADC contributed the most, with DSC, T1CE, and FLAIR following, highlighting the value of multiparametric MRI radiomics in this context (**Figure 7**).

**Figure 8:**
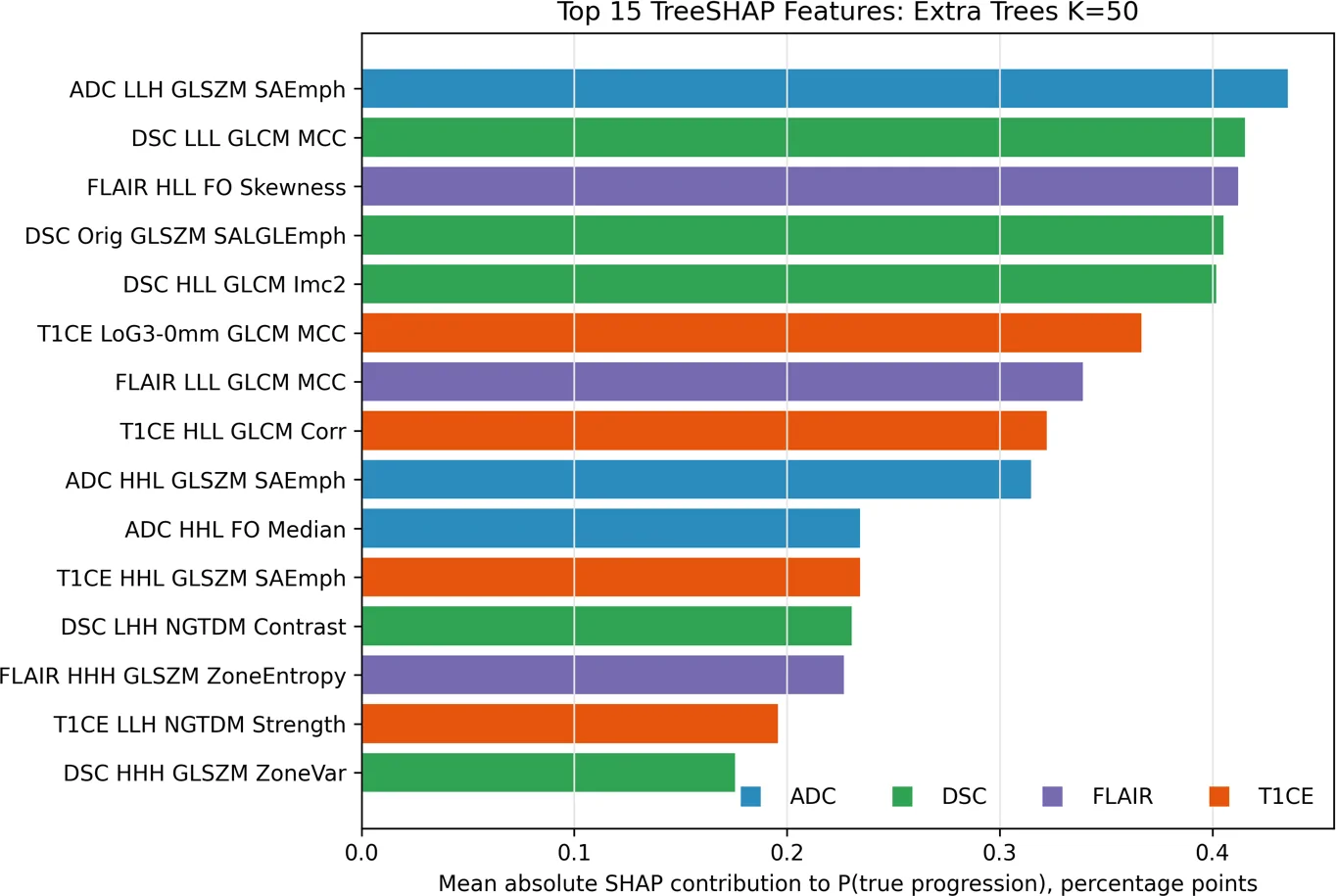
Cumulative SHAP importance for the selected Extra Trees K = 50 model. Features are ranked by mean absolute SHAP value on the held-out test set. The cumulative curve rises rapidly and approaches 80% of total attribution by approximately the top 20–25 features, indicating that a limited subset of radiomic predictors accounts for most of the model’s signal.

The most influential features included ADC wavelet LLH GLSZM SmallAreaEmphasis, DSC wavelet LLL GLCM MCC, FLAIR wavelet HLL first-order skewness, DSC original GLSZM SmallAreaLowGrayLevelEmphasis, and DSC wavelet HLL GLCM Imc2. At the feature-family level, texture features contributed most prominently, particularly GLSZM and GLCM features, followed by first-order, GLDM, and NGTDM features (**Figure 8**, **Table 5**).

**Table 5:** Top SHAP-ranked radiomic features in the validation-selected Extra Trees K = 50 model.

Rank	Feature	Meaning	Mean |SHAP|, pp
1	ADC Wavelet LLH GLSZM SmallAreaEmphasis	Small homogeneous ADC texture zones	0.436
2	DSC Wavelet LLL GLCM MCC	Complex perfusion texture co-occurrence	0.415
3	FLAIR Wavelet HLL First-order Skewness	Asymmetry of FLAIR intensity distribution	0.412
4	DSC Original GLSZM SmallAreaLowGrayLevelEmphasis	Small low-intensity perfusion texture zones	0.405
5	DSC Wavelet HLL GLCM Imc2	Nonlinear perfusion texture dependence	0.402
6	T1CE LoG 3.0 mm GLCM MCC	Edge-enhanced enhancing-tumor texture complexity	0.367
7	FLAIR Wavelet LLL GLCM MCC	FLAIR texture co-occurrence complexity	0.339
8	T1CE Wavelet HLL GLCM Correlation	Enhancing-tumor gray-level dependency	0.322
9	ADC Wavelet HHL GLSZM SmallAreaEmphasis	Small homogeneous ADC texture zones	0.315
10	ADC Wavelet HHL First-order Median	Median ADC intensity	0.234
11	T1CE Wavelet HHL GLSZM SmallAreaEmphasis	Small homogeneous enhancing-tumor texture zones	0.234
12	DSC Wavelet LHH NGTDM Contrast	Local perfusion gray-tone variation	0.230
13	FLAIR Wavelet HHH GLSZM ZoneEntropy	FLAIR texture-zone heterogeneity	0.227
14	T1CE Wavelet LLH NGTDM Strength	Local enhancing-tumor texture strength	0.196
15	DSC Wavelet HHH GLSZM ZoneVariance	Variability of perfusion texture-zone sizes	0.176

Our model integrated diffusion, perfusion, postcontrast, and FLAIR-derived radiomic information. The strongest contributions came from texture-based descriptors of spatial heterogeneity, suggesting that multiparametric radiomics captures complementary aspects of post-treatment GBM biology that are relevant to distinguishing TP from treatment-related changes.

## Discussion

We developed and validated an explainable, calibrated, multiparametric MRI radiomics model to differentiate TP from TRC in post-treatment GBM. Our model achieved strong performance in differentiating TP from TRC, with an AUC of 0.89 on the held-out test set. The implementation of probability calibration further improved the reliability of the model’s outcome predictions. Furthermore, although TP was designated as the positive class due to the higher clinical risk linked with missed active tumors, the model still demonstrated strong sensitivity and specificity. Its balanced accuracy indicates that performance was not solely driven by the majority class, suggesting that the model could provide a reliable, calibrated estimate of TP probability during post-treatment monitoring. Our results indicate that multiparametric MRI radiomics can provide valuable noninvasive insights for distinguishing recurrent tumors from TRC after chemoradiation. However, since these findings are based on a single-center retrospective dataset and require validation through multicenter studies and reader comparisons, they should be considered preliminary.

The SHAP analysis validated the biological plausibility of the model. Predictions were not driven by just one sequence; rather, all four modalities, ADC, DSC, T1CE, and FLAIR, each contributed significantly with relative attribution. This balanced contribution makes clinical sense because TP and treatment-related changes cannot be distinguished based on a single imaging characteristic. Recurrent tumors typically demonstrate increased cellularity and new blood vessel formation, which may be reflected in lower ADC values and higher perfusion, respectively. In contrast, treatment effects such as injury can exhibit diverse enhancement patterns and edema, linked to factors such as vascular permeability, necrosis, gliosis, and inflammation, which are associated with higher ADC values and lower perfusion (24, 25). Existing literature supports the biological association of diffusion radiomics, which uses ADC to distinguish recurrent GBM from radiation necrosis (26). Additionally, multiparametric radiomics models that include ADC and CBV have surpassed traditional MRI radiomics and single-parameter ADC or CBV measurements in their ability to accurately identify pseudoprogression (9). Our model shows that features from ADC and DSC sequences, along with T1CE and FLAIR attributions, indicate that radiomics captures diverse phenotypes related to diffusion, perfusion, enhancement, and edema, rather than relying on a single imaging sequence.

At the feature level, the top predictors were primarily multiscale texture descriptors rather than simple intensity measures. These features captured small homogeneous ADC zones, complex DSC co-occurrence patterns, asymmetric FLAIR distributions, and heterogeneous tumor architecture on T1CE. This profile is biologically plausible because recurrent tumors and treatment-related changes differ in regional patterns of cellularity, vascularity, enhancement, edema, necrosis, and inflammation, rather than by a single characteristic. Previous diffusion-radiomics research shows ADC features can differentiate recurrent GBM from radiation necrosis, with second-order texture features reflecting spatial variation in diffusion related to tumor cellularity, necrosis, vascular injury, and gliosis. These differences are evident on imaging, with recurrent tumors showing lower ADC and higher rCBV, whereas treatment-related changes show higher ADC and reduced perfusion (24).

Our findings align with previous radiomics research on this important clinical problem. Kim et al. (2018) developed a multiparametric MRI radiomics model using contrast-enhanced T1-weighted images, FLAIR, ADC, and CBV maps. They reported an AUC of 0.96 in the internal validation group and 0.85 in the external validation group for distinguishing pseudoprogression from early true progression (9). Similarly, Elshafeey et al. (2019), in a multicenter study, built a classifier that used Ktrans features from dynamic contrast-enhanced MRI (DCE) and rCBV from DSC, which achieved an AUC of 89.10%, sensitivity of 91.36%, and specificity of 88.24% for differentiating pseudoprogression from progressive disease in GBM (27). Lastly, Park et al. (2021) evaluated diffusion radiomics for distinguishing recurrent GBM from radiation necrosis, achieving an independent test AUC of 0.80, with 78% accuracy, 66.7% sensitivity, and 87% specificity, further demonstrating the value of diffusion-based radiomics (26). Our internal AUC of 0.887, which was held out, falls within the range reported for previous MRI radiomics methods. The high specificity for treatment-related changes is also clinically important because misclassifying treatment effects as progression could lead to premature treatment escalation, unnecessary biopsy, or resection (28). However, the optimal threshold may depend on the intended clinical application; settings that prioritize maximal TP detection may reasonably favor lower thresholds to achieve higher sensitivity, at the expense of increased false-positive classifications. At the primary Youden threshold of 0.869, the model achieved a sensitivity of 0.750, specificity of 0.923, PPV of 0.960, and NPV of 0.600. In contrast, the sensitivity-prioritized threshold of 0.582 increased sensitivity to 0.906, with corresponding specificity of 0.462, PPV of 0.806, and NPV of 0.667. This threshold reduced the number of false negatives from 8 to 3, but increased the number of false positives from 1 to 7, indicating that six additional TRC examinations would have been classified as tumor-positive disease. This illustrates the expected trade-off between minimizing missed tumor-positive cases and avoiding overclassification of treatment-related changes as tumor-positive disease (**Supplementary Table 1**).

This approach offers additional quantitative insight during GBM post-treatment surveillance. In routine settings, differentiating TP from TRC is often discussed in multidisciplinary tumor boards, especially when biopsy or reoperation isn’t feasible or appropriate. A calibrated radiomics model can provide valuable context by estimating the likelihood of TP rather than just giving a simple prediction. Decision curve analysis further assesses whether the model-derived probabilities provide a meaningful net benefit across relevant clinical thresholds. Therefore, this approach should support rather than replace neuroradiologic interpretation, RANO assessment, clinical status, corticosteroid use, treatment timing, and longitudinal imaging follow-up. Recent evidence that systemic inflammatory markers may help distinguish pseudoprogression from TP further supports the need for future multimodal models that integrate imaging radiomics with clinical and blood-based biomarkers (29). In this context, radiomics may serve as an adjunctive tool to highlight subtle multiparametric imaging patterns, support tumor board discussions, and aid interpretation of tumor versus treatment-related change.

Our study has several strengths. First, the cohort was relatively large for a post-treatment glioblastoma radiomics study. Second, the model integrated complementary anatomic, diffusion, and perfusion information from T1CE, FLAIR, ADC, and DSC imaging rather than relying on a single sequence. Third and most importantly, the study evaluated model performance beyond discrimination alone. Probability calibration assessed the reliability of predicted risks, and decision curve analysis evaluated the potential clinical usefulness across different threshold probabilities. Lastly, SHAP explainability was performed at multiple levels, including modality- and feature-level attribution. Together, these elements provide a more transparent and clinically interpretable evaluation than approaches that report AUC alone.

At the same time, this study has several limitations. First, most reference labels were assigned based on longitudinal clinical and radiologic follow-up rather than on histopathology alone. Although this reflects real-world post-treatment GBM assessment, where biopsy or repeat resection is often not feasible or appropriate, it may introduce residual label uncertainty. Because model performance and calibration were estimated against these inherited dataset-defined labels, any misclassification in the reference standard could affect sensitivity, specificity, and probability calibration. Second, as a retrospective study based on a single dataset, the findings may have limited applicability across different institutions, scanners, protocols, segmentation methods, and treatment approaches. Third, although we separated patient data for training/internal validation and an internal test set, we did not conduct external validation. Thus, the model should be viewed as internally validated and hypothesis-generating rather than ready for clinical use. Fourth, the smaller size of the TRC group relative to the TP group could affect the stability of threshold-dependent metrics, calibration, and subgroup analyses. Fifth, radiomic features can be affected by preprocessing, image normalization, segmentation quality, and variations in MRI acquisition. Because operator-specific metrics were unavailable, a quantitative assessment of mask quality was not feasible. Finally, our radiomics approach was based on whole-lesion segmentation, reflecting a pragmatic design aligned with routine clinical imaging interpretation and radiology workflow. However, we acknowledge that compartment-specific analyses, particularly of perfusion-derived features, may reveal additional biologically relevant heterogeneity in post-treatment glioblastoma and should be explored in future studies. Additionally, future research should validate this method in external multicenter cohorts, assess its robustness across various scanners and segmentation approaches, and prospectively determine whether calibrated, explainable radiomics can enhance standard multidisciplinary evaluations.

In summary, distinguishing TP from TRC remains a central challenge in post-treatment GBM surveillance because both entities can present with overlapping enhancement and FLAIR abnormalities on conventional MRI. In this internally validated, single-source retrospective study, a multiparametric MRI radiomic model incorporating T1CE, FLAIR, ADC, and DSC features showed promising held-out performance, with good discrimination, high specificity for treatment-related change, and improved calibration after Platt scaling. Exploratory decision curve analysis suggested potential net benefit, but these findings should be interpreted with caution, given the limited test set size and the small number of TRC cases. SHAP-based attribution provided a transparent description of the imaging modalities and radiomic features that contributed to the model’s predictions, although these findings should be viewed as model explanations rather than biological validation. Our modeling approach may provide complementary quantitative information for post-treatment GBM assessment, but the results remain hypothesis-generating. External multicenter validation across scanners, acquisition protocols, preprocessing pipelines, segmentation methods, and reference-standard strategies is required before clinical implementation.

## Data Availability

Publicly available datasets were analyzed in this study. This data can be found here: The Cancer Imaging Archive (TCIA): UCSD-PTGBM collection. Direct link: https://www.cancerimagingarchive.net/collection/ucsd-ptgbm/.
