# Supplementary material for: Calibrated and explainable multiparametric MRI radiomics for differentiating tumor positive disease from treatment-related changes in glioblastoma
Source: Front Oncol. 2026 Aug 18;16:1890956. doi: 10.3389/fonc.2026.1890956 (PMC13529537; doi:10.3389/fonc.2026.1890956)
Supplement: Supplementary file 1 [file DataSheet1.docx]

**Figure S1.** *Study cohort flow diagram. Flowchart showing cohort selection, exclusions, final analytic cohort, examination-level class distribution, and patient-level train/test split.Note. TP = tumor positive; TRC = treatment-related change. Some excluded patients contributed other eligible examinations that remained in the final cohort.*

*Figure S2 Bootstrap stability of train-only radiomic feature selection across 200 patient-level resamples. Individual-feature stability was moderate, whereas modality- and feature-family-level stability showed consistent selection of multiparametric and texture-based radiomic information.*

***Figure S3.*** *Learning-curve analysis of the selected Extra Trees model using patient-grouped cross-validation. OOF ROC-AUC and PR-AUC increased with larger training fractions and plateaued between 80% and 100% of training patients, suggesting diminishing performance gains with additional data.*

**Supplementary Table S1. Comparison of threshold strategies for the selected Extra Trees model on the independent held-out test set.**

| **Threshold strategy** | **Probability threshold** | **Sensitivity** | **Specificity** | **PPV** | **NPV** | **TP** | **TN** | **FP** | **FN** |
| --- | --- | --- | --- | --- | --- | --- | --- | --- | --- |
| Youden threshold | 0.869 | 0.750 | 0.923 | 0.960 | 0.600 | 24 | 12 | 1 | 8 |
| Sensitivity-prioritized threshold | 0.582 | 0.906 | 0.462 | 0.806 | 0.667 | 29 | 6 | 7 | 3 |
